# A map of the class III region of the sheep major histocompatibilty complex

**DOI:** 10.1186/1471-2164-9-409

**Published:** 2008-09-11

**Authors:** J Qin, C Mamotte, NE Cockett, JD Wetherall, DM Groth

**Affiliations:** 1 School of Biomedical Sciences, Curtin University, Perth, 6845, Western Australia; 2 Department of Animal, Dairy and Veterinary Sciences, Utah State University, Logan, UT 84322-4700, USA

## Abstract

**Background:**

The central, or class III, region of the major histocompatibility complex (MHC) is an important gene rich sub-region of the MHC of mammals and contains many loci implicated in disease processes and potential productivity traits. As a prelude to identifying MHC loci associated with productivity traits in sheep, we have used BAC and cosmid libraries of genomic DNA to generate a physical map of the sheep MHC class III region. This map will facilitate association studies and provide insights into the distribution of recombination events in this chromosomal segment.

**Results:**

Twenty eight sheep genes were identified in 10 BAC clones which spanned approximately 700 kbp of a chromosomal region adjacent to the class I region of the sheep MHC and which therefore covers most, if not all, of the class III of the sheep MHC. The relative positions of 17 of these genes was established as well as two additional groups of genes for which the intragroup order was not known. Cosmid mapping permitted a more detailed mapping of the complement genes present in the class III and showed a local inversion (relative to humans) of one pair of the duplicated complement C4 and CYP21 loci. A panel of 26 single nucleotide polymorphisms (SNPs) was identified in 10 loci, covering ≈600 kbp of the mapped region.

**Conclusion:**

This report provides a physical map covering ≈700 kbp of the class III of the sheep MHC together with a SNP panel which will facilitate disease and productivity association studies. The presence of a local inversion (relative to humans) of one pair of the duplicated C4 and CYP21 loci and a previously described dinucleotide tandem repeat locus (BfMs) has been located within an intron of the SK12VL gene.

## Background

The major histocompatibility complex (MHC) consists of a cluster of genes within a genomic region which encodes molecules responsible for the expression of adaptive and innate immune responses in vertebrates [1]. The human MHC is the most gene-dense region of the human genome [2,3]. Mammalian MHC typically comprise three regions – class I and class II regions usually separated by a central region which is sometimes referred to as the class III region [4]. In some species the class I and class II regions are further subdivided to include an extended class I and extended class II region respectively. The class I and class II regions contain genes encoding glycoproteins expressed predominantly by cells of the immune system and which act as receptors for peptides derived from self and non-self antigens [5].

The MHC class III is very gene dense and contains genes of many types including the complement genes C4, C2 and CFB, the Hsp70 genes and genes in the TNF cytokine family. Many other genes not directly associated with immune responses have also been mapped to this region [6]. Loci within the class III are often highly conserved and in general terms evolved prior to the class I and II loci [4,3].

In humans, the class III spans approximately 800 kbp with a gene density of one per 11.4 kbp [7]. The class III is sparse in pseudogenes relative to the class I and II regions, although some such as the complement C4, CYP21A and TNXB pseudogenes, have been well studied in certain haplotypes [2]. Moreover, some genes such as TNXB and CYP21B display overlaps in mRNA transcription with other genes [4].

The extreme polymorphism and numerous disease associations of loci within mammalian MHC have led to much research on this genomic region, including in species of economic importance such as ungulates. Relative to other mammals, the sheep and cattle MHC are poorly characterized. Paterson and colleagues [8] have shown that the broad structure of the sheep MHC region appears to be similar to its human counterpart with a class III separating class I and II regions. More recently Liu and colleagues [9] used non-proprietary BAC clones to provide a broad description of the sheep MHC and confirmed that a class III flanked by class I and class II regions existed which contained loci orthologous with those present in the human MHC class III. This work proves a provisional framework for the more detailed mapping of this region.

Several microsatellite loci have been mapped to the sheep MHC; microsatellites occur within the OLADRB and OLADRBps loci in the class II region [10,11] while OMHC1 [12] is a dinucleotide microsatellite within the class I region and the BfMs microsatellite locus [13] maps close to the CFB locus. In this report we describe an ordered BAC contig map of the MHC class III based on characterization of clones from the CHORI-243 sheep BAC library identified by comparative analysis of loci typically present in the class IIIs of the human and mouse MHC. A physical map covering ≈700 kbp of the sheep class III was generated together with a panel of SNPs. Both should contribute to the identification of partial MHC haplotypes in sheep which can be used for disease association studies and to provide insights to subregions within the sheep MHC.

## Results

### Identification of BAC clones containing sheep class III MHC loci

**Figure 1:**
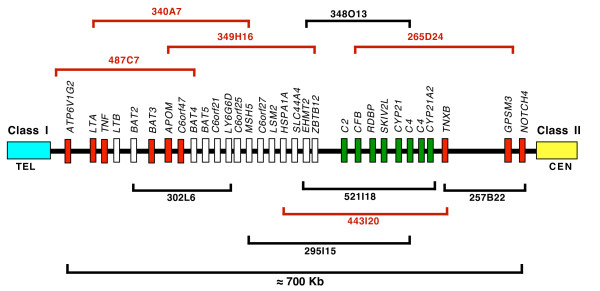
**Physical map of the class III of sheep MHC**. The relative order of 10 BAC clones spanning ≈700 kbp containing sheep orthologues of loci mapped to the class III of the human MHC. The presence of the class I region was confirmed by the presence of a class I locus in BAC clone 487C7. The start of the class II region was inferred from the location of the Notch4 gene in BAC 257B2. Individual loci (31) are shown as vertical bars; the distances between bars are not to scale. Bars shaded red (9) and green (8) depict loci for which the relative positions are known; red bars are derived from sequencing BAC clones while green bars are derived from sequencing of cosmid clones. The open bars comprise two groups of 14 loci for which the relative position is known but for which the intragroup order of loci is not known. The map of these loci has been inferred assuming a similar map to that of the human MHC.

The initial screening of the CHORI-243 sheep BAC library with radiolabelled locus specific overgo probes for 10 putative orthologous loci that cover the entire length of the human MHC class III identified 89 positive colonies. Following a second screening with PCR primers of sheep origin specific for these 10 loci, 34 of the 89 BAC clones generated amplicons of predicted sizes. Ten of these BAC clones were selected for further characterisation based upon both the number of loci present per clone and the availability in GenBank of end sequences which anchored at least one end of the clone in a known gene. Subcloning and partial sequencing of 5 BAC clones, plus the information obtained from the end sequences of the remaining five clones, permitted the assembly of the contig map shown in Figure 1. This map covers ≈700 kbp anchored by a clone (487C7) manifesting a 5' end sequence homologous with a human class I sequence and several classical class III loci thereby identifying one end of the sheep class III. Another clone (257B22) contained a 3' sequence homologous with the Notch4 gene which is close to the start of the class II region in the human MHC.

### Cosmid mapping of the MHC associated complement genes in sheep

**Figure 2:**
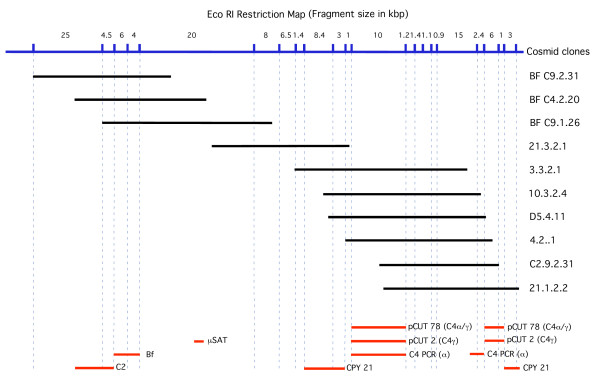
**Molecular mapping of MHC linked complement genes based on analysis of cosmid clones**. A molecular map of MHC associated complement genes and steroid 21 hydroxylase loci based on EcoRI restriction pattern analysis of clones from a male sheep cosmid library. DNA (≈10 mg) from each clone was digested with EcoRI and size fractionated on 0.7% agarose gel and stained with ethidium bromide. The gel was then blotted onto Hybond N+ nylon membranes by vacuum assisted capillary blotting under alkaline conditions (0.2 M NaOH, 0.4 M NaCl). Following depurination, the membranes were then sequentially hybridised with probes in 5 × SSPE containing 7% SDS, 1% BSA and 5 mM EDTA and subsequently washed at elevated stringency (0.1 × SSC, 0.1% SDS, 65°C). Fragment sizes were determined from a 1 kb ladder (Bethesda Research Laboratories).

Initial screening of approximately 100,000 colonies of the cosmid library with the complement C4 pCUT78 probe (specific for part of the a chain and all the g chain regions of cattle C4 cDNA) at high stringency resulted in the identification of three positive clones (3.3.2.1, 4.2.1 and 10.3.2.4) each of which manifested distinct fragment patterns following digestion with EcoRI. A terminal 2.3 kbp EcoR1 fragment from cosmid 10.3.2.4 was chosen as a probe to rescreen the library for overlapping clones which extended the chromosomal region covered by three additional cosmids that contain the sheep CFB and C2 loci. The contig map derived from these patterns is shown in Figure 2.

A Southern blot of independent cosmid clones containing complement C4 sequence were Southern blotted and hybridised with several C4 specific probes to ascertain the length of genomic DNA (Figure 2). As expected, the pCUT78 probe hybridised to all cosmid clones and identified a 10.1 kbp DNA fragment. However clone C2.9.2.31 contained a 5.0 kbp C4 positive fragment but lacked the C4 positive 10.1 kbp fragment thereby demonstrating the presence of at least two distinct C4 loci. Clones 4.2.1 and D5.4.11 contained two fragments that hybridised with a pCUT78 probe indicating that they contained both C4 genes. A probe generated by PCR of sheep DNA and specific for the C4 alpha chain [14] showed that the pCUT78 positive 10.1 kbp fragment hybridised with the C4 alpha chain probe. However clones 4.2.1 and D5.4.11 contained an additional 2.4 kbp C4 alpha chain positive fragment and clone C2.9.2.31 contained two positive C4 alpha chain fragments (2.4 kbp and 1.5 kbp). These data indicated the transcriptional orientation of the C4 gene (right side of Figure 2).

Hybridisation of the Southern blot with the human CYP21A probe revealed the presence of two cross hybridising sheep loci. One of these was present on a 3.0 kbp fragment at the end of cosmid 21.1.2.2 and therefore has a similar transcriptional orientation as the adjacent C4 gene. The other CYP21 gene is present in an 8.4 kbp fragment in cosmids C2.3.4.57 and 3.3.2.1 which do not contain the 3.0 kbp fragment identifying its paralogue. These results indicate that in sheep both the C4 and CYP21 loci are duplicated and that their relative order is ..CYP21..C4..C4..CYP21..

Hybridisation of cosmid Bf4.2.20 with C2 and CFB specific human probes revealed the presence of both loci in this cosmid and hybridisation with a radiolabelled probe specific for the BfMs microsatellite locus showed that it was located to the right of the CFB gene in this cosmid. Subsequent direct sequencing of this cosmid revealed a partial sequence of sheep C2 (≈5000 bp) followed by a complete sequence of CFB (≈5000 bp) and a partial sequence of the RDBP gene (≈376 bp). These loci are very close to each other with approximate intra-locus distances of 600 bp and 4 bp respectively. Thus the map for this subregion is ..C2.(600 bp).CFB.(4 bp).RDBP. BAC clone 52118 contains the C2, CFB, SK12VL, C4 and CYP21 loci. Direct sequencing showed that the BfMs microsatellite locus was located within an intron in the SK12VL gene which was adjacent to a CYP21 gene. This information has been summarised in Figure 1.

### Detection of SNPs

Ten of the loci that covered the predicted span of the sheep class III (Figure 1) were amplified and the amplicons sequenced from genomic DNA from at least five individual sheep using primers shown in Table 1. Locus specific polymorphisms were observed and a panel of 26 SNPs was generated (Table 2).

**Table 1:** PCR primers from sheep sequences identifying locus specific SNPs.

Locus	Forward Primer (5'-3')	Reverse Primers (5'-3')
APOM	GGTGGGTAGATTAGGGAGTC	CACACTGGCTATGTTGACAC
BAT2	AGCGAGGAATACTTCTTTCC	GCTGAGCAAGCTCATAAAAT
BAT5	GTTTGAGCACCTTCTCTCTG	ATGGTGAGGGTTAAATTGG
C2	GACCCAGAAAGTGAAGATGT	ACAGAAGGATGGAGTGAGTG
C2	GTTCCCTACCCCACAGAT	CTGGGCCATTGTAGTTTTAC
G6B	CCTAGAAGAGGGAGAAAAGC	GGATCCTTGAGTCTAGTGGA
LTA	TTTTCCAAACCAAGACACTT	TAGAAGATGCTGCTGTTTCA
MSH5	GGAGCCTCCTAAGATTTGTC	AACACAATCACAAGACAGCA
MSH5	GTCCTGAGCAAATCAGTCAT	ACCTCTGATGATGAAACTGG
MSH5	TTGCAAAGAGTTCACACAAC	CAGGTGACAGCTCTAAGCA
NG22	TTGTGAGGGTGGTGATAAAT	GATGCTCTGGGAGATAGATG
TNXB	ACCATCACCACAACAAAGAT	GGACCTTGAAGGAGTCAAAT
TNF	GAGCGGAGGTTCAGTGAT	GGGTTCTTACCGGAATACTT

**Table 2:** SNP panel in sheep MHC class III region

Locus Name	GenBank ID	Mutation	Position	SEQUENCE
APOMS1	EF139192	G/A	1423	TTGGCAGGAC(G/A)GCCAGCTCAT
BAT2S1	EF197828	C/T	1441	AAACAGCAAG(C/T)CCAAGCCCTA
BAT2S2	EF197828	A/G	1101	TCCCTGCTCC(A/G)CCAAGGGCCT
BAT5S1	EF197830	A/G	225	GCTTGGGGAC(A/G)GGGGTACCCT
BFS1	EF446375	C/T	2922	CACTCCATGA(C/T)CTCTCACTCA
BFS2	EF446375	C/T	4222	CCATTCTGAG(C/T)TTCTGCAGTG
C2S1	EF446374	A/G	1468	CACGGTGAGC(A/G)CTGGACTCAG
C2S2	EF446374	G/C	1809	CTAGCAAGCT(G/C)TGCCCTGGGC
C2S3	EF446374	C/T	1394	GGCCTATCTG(C/T)CTCCCCTGCA
C2S4	EF446374	C/T	320	GAGCGGAGGG(C/T)ATCCTGCTCA
G6bS1	EF197833	C/T	1396	AGGAGTTGAG(C/T)GACCTACATT
LTAS1	DU475543.1	A/G	259	CAGGTGGGAG(A/G)GTATATACTG
LTAS2	DU475543.1	C/T	486	CCTTCTCCTC(C/T)GAATTTACTG
LTAS3	DU475543.1	A/G	254	TGTCCCAGGT(A/G)GGAGGGTATA
JQMSH5S1	EF197839	A/T	3781	TATTTTAGAA(A/T)GAAGAATAGT
JQMSH5S2	EF197839	A/G	3844	ATGCAATGCT(A/G)CAGGTTAAAG
JQMSH5S3	EF197839	C/T	2169	GACAAAATTA(C/T)GGAATGTTCC
JQMSH5S4	EF197839	A/G	2832	TTCTGCTCCA(A/G)TTGGTCCATG
JQNG22S1	EF197840	A/G	926	GTACGGTGCC(A/G)GAGCTCCCAG
TNXBS1	EF197845	C/T	1293	CTCTACCTGT(C/T)TCTCAGAACC
TNXBS2	EF197845	A/G	1324	GAAGAAATCG(A/G)AGCAGAGCCT
TNFaS1	EF446377	A/G	2408	GCCTCCTTTT(A/G)CTTATGTTTT
TNFaS2	EF446377	A/G	2149	CTTCCTGCCA(A/G)TGTTTCCAGA
TNFaS3	EF446377	A/G	1590	CTGCCATCAA(A/G)AGCCCTTGCC
TNFaS4	EF446377	A/G	1389	GGGGGGACTC(A/G)TATGCCAATG
TNFaS5	EF446377	C/T	1951	GCCTGGACAA(C/T)GGGCCACCAA

Location and allelic variation of sheep MHC class III SNPs with GenBank accession numbers.

## Discussion

The two approaches used in this work permitted the construction of a physical map of the sheep MHC class III region. First, a broad contig map based on analysis of BAC clones was initially generated. Secondly, characterisation of cosmid clones (identified in an earlier unpublished study) from a sheep genomic library permitted identification and more detailed mapping of the region containing the C2, CFB and C4 complement genes and the CYP21 sex steroid hydroxylase loci. In addition a panel of SNPs was generated representing 10 loci spanning the sheep MHC class III region.

The 10 BACs used in this study which span ≈700 kbp may represent most of the class III region although the boundary with the class II region remains to be confirmed by characterisation of a BAC clone containing both class II region genes and class III loci. For example, Notch 4 is known to be at the class II end of the human class III and this work therefore extends the sheep class III beyond the 600 kbp reported by Liu and colleagues [9]. The final map produced contains 17 loci for which the relative positions are known plus two groups of loci for which the relative position is known but for which the intragroup order of loci is not known. It is hypothesised that their order will be similar to the corresponding syntenic group in the human MHC class III. These data show that the class III regions of the sheep and human MHC are very similar with respect to length, location and orientation between the class I and class II regions, and their gene composition. This result differs from the map reported by Liu and colleagues [9] for the cattle MHC class III region for which the class I end contains a duplicated and inverted subregion of unknown length which places an additional C4 gene adjacent to the class I region. Inspection of the latest version (version 4) of the cattle genome assembly (UCSC Genome Bioinformatics – ) does not support the rearrangement proposed by Liu and colleagues [9] but does support the broad map reported in this study.

Detailed analysis of cosmid clones containing the complement C4, C2, and CFB genes plus the CYP21 loci revealed a localised inversion of this sub-region such that the order of these genes in sheep is C2..CFB..RDBP..SK12VL..CYP21..C4..C4..CYP21 ..class II while in the well characterised human class III the order is C2..CFB..RDBP..SK12VL..C4A..CYP21A..C4B..CYP21B(pseudo)..class II. The mapping data do not show which pair of C4/CYP21 loci has inverted relative to the human order, although the transcription of the furthermost C4 locus suggests that it is the CYP21 and C4 loci closest to CFB that have transposed. Additional sequencing will be required to confirm this prediction. Although it is clear that the sheep has two CYP21 loci, it is not known if they are functional. The cosmid clones covered approximately 115 kbp of sheep DNA and provide a detailed map of this region. Interrogation of version 4 of the cattle genome assembly (UCSC Genome Bioinformatics – ) shows that a similar local rearrangement of C4 and CYP21 loci exists in cattle, although the assembly lists three C4 loci whereas only two such loci were detected in sheep. This could reflect a species difference, or even an individual animal variation, since copy number variation of C4 loci has been observed in several mammalian species. The cosmid mapping showed the presence of the previously described BfMs microsatellite locus close to the CFB locus. Sequencing of a DNA fragment subcloned from a BAC clone (443I20) revealed that this polymorphic locus is actually located within the 5' end of the SK12VL gene which is itself ≈729 bp downstream from the 3' end of the CFB gene.

Dalrymple and colleagues [15] constructed a virtual map of the sheep genome based largely on end sequencing of the CHORI-243 BAC library used in this study, including structural comparisons and synteny with the better characterised genomes of other mammals. The virtual map published by this group for the MHC region is consistent with the mapping data determined in this study. Another extensive study published recently by Wu and colleagues [16] utilized radiation hybrid mapping to provide a map of sheep chromosome 20 which includes the MHC. The partial map of the central region reported by these workers shows discrepancies with both this study and the comparative information in version 4 of the cattle genome assembly.

Although this study shows structural similarity between the human and sheep MHC, it is known that the MHC of mammals exhibit considerable structural diversity and it is likely that further studies will show species specific differences between the class IIIs of the sheep MHC and those of other species including the human MHC. The localised inversion of the C4 and CYP21 loci in sheep described in this report is one example of this type of diversity. Our map is the result of analysis of DNA from two male sheep (originators of the BAC and cosmid libraries respectively). It is known that in humans there is significant haplotype diversity, especially in the complement containing subregion of the class III region, which is believed to reflect the ancestral haplotypes and block-like structure of this genomic region [17]. Hence it is likely that individual sheep will manifest structural diversity in this region. However this will only become apparent when analysis of SNPs, such as those reported in this study, are used to asses haplotype diversity.

## Conclusion

This report provides a physical map covering ≈700 kbp of the class III of the sheep MHC together with a SNP panel which will facilitate disease and productivity association studies and permit studies of block-like subregions within this region. The map was generated using clones from the CHORI-243 sheep BAC library and from a cosmid genomic library from Clontech. The sheep class III region is remarkably similar to the human class III, although a local inversion of the duplicated C4 and CYP21A/B loci relative to humans is a significant point of difference. A more extensive inverted duplication reported by Lie and colleagues [9] for the cattle MHC class III was not observed in sheep. A previously described dinucleotide tandem repeat locus (BfMs) has been located within an intron in the SK12VL gene.

## Methods

### Strategy for identification of sheep MHC class III orthologues of human and mouse genes

Primers amplifying sheep orthologues of human and mouse MHC class III genes were derived from exonic regions of an alignment of genomic human sequence with its orthologous mouse cDNA sequence. The loci chosen and the primers identified are summarised in additional file 1. These primers were used to amplify sheep DNA and the amplicons were cloned into the pGEM-T easy vector and sequenced. The resulting sheep sequences, together with the pairs of overgo primers derived from these sequences which were used to screen the CHORI-243 sheep BAC library, are summarised in additional file 2. Overgo primers were designed using the Overgo Maker Program .

During the course of this study end sequences of many of the BAC clones of the CHORI-243 library were made available in GenBank. Five of these sequences permitted identification of additional genes within the sheep MHC class III while the other five BACs were subcloned and re-screened for specific loci. Finally a contig map was generated from analysis of the 10 BAC clones which spanned ≈700 kbp of the putative class III of the sheep MHC. This map was the result of identifying genes in sequences derived from 5 subcloned BACs and identification of genes in the other 5 BACs with published end sequences. The accession numbers of the 10 BAC clones used in this study are summarised in Table 3.

**Table 3:** GenBank accession numbers for the end sequences of the ten BAC clones in Figure 1.

Clone ID	GenBank Acc No (gi)
**CH243-257B22**	77151234/77143173>
CH243-265D24	77125512/77125520
**CH243-295I15**	77241001/77237208
**CH243-302L6**	77296914/77298047
CH243-340A7	77275575/77263741
**CH243-348O13**	76832624/76828474
CH243-349H16	76828020/76826237
CH243-443I20	76938294/76938211
CH243-487C7	not available
**CH243-521I18**	77339223/77338595

Normal font: BACs subjected to additional direct sequencing for confirmation and identification of specific loci. **Bold **font: BACs for which published end sequences in GenBank contained orthologues of genes from the human MHC class III.

In a parallel but earlier study, a sheep (male) genomic cosmid library (Clontech) was screened with several probes to identify cosmid clones containing the complement gene region of the sheep MHC. Direct sequencing of cosmid BfC4.2.20 resulted in the identification of the C2/CFB subregion.

SNP discovery was performed by designing primers from exonic regions of sequenced loci which amplified ≈500 bp. SNPs were then identified from alignments of sequences from at least five unrelated animals or occasionally by alignments with corresponding sequences from GenBank. SNPs were confirmed when at least two heterozygote genotypes and one of each homozygote genotype were observed.

### Labelling of DNA and oligonucleotide probes

The Prime-a-Gene^® ^Labelling system (Promega) was used to label probes generated from cloning of PCR amplicons. A standard labelling reaction containing 25 ng DNA probe was prepared according to the manufacturers' protocol. The reaction was incubated at RT for 1 hour.

### Labelling overgo overlapping oligonucleotide probes

Overgo primers were labelled using the method of Gustafson *et al*. [17] (2003) with minor modifications. Briefly, a 10 μl reaction was set up for each probe comprising 1 μl each of 10 μM forward and reverse primer, 1 μl each of 250 μM dTTP and dGTP, 1 μl of 3000 Ci/mmol each of ^32^PαdATP and ^32^PαdCTP, 1 μl (2U) Klenow fragment DNA polymerase, 1 μl 10× buffer and 2 μl hpH_2_O. After incubation at 37°C for 30 minutes, 1 μl each of 250 μM dATP and dCTP was added and incubated for another 15 minutes. Following incubation, reactions for all probes were pooled and purified through a Sephadex G-15 gravity column to eliminate unincorporated nucleotides.

### BAC and cosmid library screening

Six high-density replica filters each containing 18,000 distinct BAC clones from the CHORI-243 sheep BAC library (PACBAC Resources) were probed with ^32^P labelled overgo primers for the presence of loci known to be within the class III of the human MHC. The overgo primers were radioactively labelled using overgo technology [18] and hybridised in a buffered solution containing 20 × SSPE, 1%BSA, 7%SDS and 0.5 M EDTA, and incubated at 53°C overnight, and washed in 1 × SSC buffer containing 0.1% SDS. The hybridized filters were sealed in plastic and exposed to Kodak x-ray film over intensifying screens at -80°C for 2 days prior to development of the film.

Overlapping cosmid clones in the pWE16 vector containing sequences from the complement C4 gene(s) were selected from a sheep genomic cosmid library (Clontech, USA) prepared from the liver of a single male. These were identified by cross hybridisation with a 2.1 kbp bovine C4 cDNA probe designated pCUT78 [GenBank:U16749]. This cDNA probe includes the region encoding the alpha/gamma chains of bovine C4 protein and cross-hybridises at high stringency with sheep C4 genes. DNA isolated from eleven cosmid clones containing sheep C4 DNA sequences extending over about 50 kbp were digested with EcoRI, electrophoresed in 0.7% agarose gel and blotted to a Hybond N+ membrane under alkaline conditions. These membranes were hybridised sequentially (5 × SSPE, 7% SDS, 1% BSA, 5 mM EDTA and washed at elevated stringency (0.1 × SSC, 0.1% SDS, 65°C)) with a family of probes specific for the loci of interest.

DNA probes used in this study included a pCUT78 probe [GenBank:U16749], a C4 alpha chain specific probe generated by PCR of sheep DNA described by Ren et al. [14], human probes for CFB and C2 (gift of Dr Mike Carroll) and a CYP21A cDNA probe of human origin [ATCC:57420].

### Subcloning of the BAC clones

BAC DNA was extracted from colonies(QIAGEN – Large-construct Kit), digested with EcoR1 and BamH1 respectively and subcloned into the pGEM-4Z vector (Promega Life Science) following the manufacturer's instructions. Positive plasmid colonies were identified after being PCR amplified using M13 primers and size compared on agarose gel.

### Restriction analysis of cosmid clones containing complement and CYP21 genes

Cosmid clones (≈40 kbp insert) gave many fragments when digested with EcoR1. In order to determine which of these fragments was on one end of the insert DNA fragment a special property of the vector was ultilised. The vector (pWE16) has both T3 and T7 RNA polymerase promoter sequences flanking the polylinker region into which the sheep DNA was ligated. DNA from the cosmid clones was digested with EcoRI and at 10 min intervals a 10 ml aliquot of the digest was removed and further digestion terminated by the addition of 1 ml of 0.5 M EDTA. Three aliquots (at 10 min, 20 min and 30 min respectively) together with completely digested DNA were electrophoresed in agarose gel (0.7%), Southern blotted onto Hybond N+ and hybridised with ^32^P kinase labelled T3 RNA promoter oligonucleotide. Using this strategy, one end of all inserts was identified thereby enabling the relative positions of all fragments to be deduced.

### DNA Sequencing

Sequences were generated from both directions using ABI Big Dye chemistry, a 3730 DNA Analyser and M13 primers. The remainder of the genes was sequenced sequentially in both directions by walking. Sequence contigs were generated using Vector NTI contig express (Invitrogen). The identification of genomic DNA sequences was performed using BLAST software and the GenBank databases, while the intron/exon organization of genes was derived using servers for Twinscan  and GAP  and also DNA Strider software [19].

### SNP discovery and genotyping

Oligonucleotide primers (Table 1) were designed from sheep DNA sequences of genes spanning the entire sheep MHC class III so that approximately 500 bp fragments were amplified. PCR products generated from at least five randomly selected unrelated Australian Merino sheep were sequenced. SNPs were confirmed when at least two heterozygotes and one of each homozygote were observed. SNPs were genotyped by PCR and pyrosequencing using standard protocols with Pyro Gold reagents and the PSQ™ 96MA System (Biotage). In a few instances SNP genotypes were confirmed by restriction digestion of PCR amplified DNA fragments followed by 2% agarose gel electrophoresis.

## List of abbreviations

≈: approximately; C2: complement component C2; C4: complement component C4; CFB: complement component factor B; CYP21: steroid hydroxylase gene(s) – in humans the CYP21A locus is a pseudogene and its paralogue is designated CYP21B; MHC: major histocompatibility complex; RDBP: reverse direction binding protein gene; RT: room temperature; SNP: single nucleotide polymorphism.

## Availability and requirements 

:

An interactive analysis and annotation tool for finding genes in genomic sequences

:

This site contains the reference sequence and working draft assemblies for a large collection of genomes, including the cattle genome which is referred to in our paper. It has extensive visualization tools.

:

Provides access to the Twinscan software packages for various operating systems (including Mac OSX) which facilitate gene structure prediction. Also provides a link to the Twinscan N-Scan interactive web server which is currently under repair.

:

Provides access to the Washington University School of Medicine Genome Sequencing Center ToolKit page which includes software for the identification of overgo oligonucleotide primers for genome sequencing.

## Authors' contributions

JQ and DMG carried out the molecular genetic studies and DNA sequencing. JQ, DMG and JDW participated in the sequence alignments and prepared the manuscript. CM assisted with the SNP discovery. NC provided the BAC library and technical advice. DMG and JDW conceived of the study and participated in its design. All authors read and approved the final manuscript.

## Supplementary Material

Additional file 1 List of consensus primers from human and mouse exonic sequences used to generate sheep amplicons for use as probes to screen BAC clones. Click here for file

Additional file 2 Locus specific overgo primers used to identify sheep genes in BAC clones. Click here for file
