# Supplementary material for: A map of the class III region of the sheep major histocompatibilty complex
Source: BMC Genomics. 2008 Sep 11;9:409. doi: 10.1186/1471-2164-9-409 (PMC2566321; doi:10.1186/1471-2164-9-409)
Supplement: Additional file 1 — List of consensus primers from human and mouse exonic sequences used to generate sheep amplicons for use as probes to screen BAC clones. [file 1471-2164-9-409-S1.doc]

### Additional file 1 – List of consensus primers from human and mouse exonic sequences used to generate sheep amplicons for use as probes to screen BAC clones.

Microsoft Word (.doc) table

| Locus | **MHC region** | **GenBank**  **Acc No** | **Primer (5′to3′)** | **Product**  **Size (bp)** |
| --- | --- | --- | --- | --- |
| Cat56 | class I | 18181758(h)  28193528 (m) | F: ggtattcttgayaagagaccagc  R: cagrggtcctccaggatcaa | 251 |
| **G7c** | central | 18673924 (h)  7381104(m) | F: ctctgcgttttgarccata  R: ccatgctctccccaacaat | 112 |
| **Bat3** | central | 23093110(h)  33147081(m) | F: GTTATYCACCTGGTGGAACG  R: GGAAGATTGAAGGTTCCAAC | 181 |
| **NG36** | central | 4529886(h)  3986763(m) | F: TTCATAGCTCTTTGGGGGACA  R: CCATCTCCCTCAAGRYTCTC | 190 |
| **G6D** | central | 4337095(h)  51243039(m) | F: ACAGTCCTGGCAAGAGMCAG  R: CCCATCATTGCAATCAAGTGG | 169 |
| **NTH4** | central | 20152668(h)  6754873(m) | F: TATGAGGGACAGAACTGCTCA  R: CTGTGTGYCCAGGCAGACACT | 241 |
| **TNFα** | central | 4337095(h)  7305584(m) | F: ACTTTATTTCTCGCCACTG  R: YGTGAAAACGGAGCTGAAC | 116 |
| **BAT4** | central | 4337095(h)  34784641(m) | F: GCTCACCTGCTGTCACTGTC  R: AGTCAGAACTCGAGGTTCATGT | 309 |

All primers were ordered from GeneWorks Pty Ltd. Mixed base codes: R(AG) Y(CT) M(AC).
