# Supplementary material for: A map of the class III region of the sheep major histocompatibilty complex
Source: BMC Genomics. 2008 Sep 11;9:409. doi: 10.1186/1471-2164-9-409 (PMC2566321; doi:10.1186/1471-2164-9-409)
Supplement: Additional file 2 — Locus specific overgo primers used to identify sheep genes in BAC clones. [file 1471-2164-9-409-S2.doc]

### Additional file 2 – Sequences of sheep amplicons and the locus specific overgo primers derived from them used to identify sheep genes in BAC clones

Microsoft Word (.doc) table

**DNA sequences of sheep amplicons from which the overgo primers used to screen the BAC library were derived. The sequence in red font were generated the overgo primers shown in the table below.**

| Locus | Sheep DNA Sequence |
| --- | --- |
| BAT3 | GGAAGATTGAAGGTTCCAACCACGACATAGCTGTTGGCATTCCGGTCAT  GAACAGAGGCCCCAGGCCCCGAGTACCAGGCGTGGGTCCCCCACCATG  GGTGCTGAGGTGGACCCTGTTCCAGAAGATGCTCCGGAAGGGAGCTGA  GTCTGAGGAGGAGCCCGTTCCACCAGGTGAATAACA |
| **Bat4** | GGAATTCACTAGTGATTAGTCAGAACTCGAGGTTCATGTATGTCCGCAG  ATCCCGTCCCAGGCCTTGTCCTTCTCTTCCTGCCTTTTCTCCTCCCTGCGG  TTCAGTGTGGTCACCCTAGGGGCTCTCTCCCTCCCAGCCACTGCCCGTAT  ATCCCGAGCTGGGAAACGTGTAACTCGGGGCTGAGGTGCTGGTCTGTAG  CCTAACCCCTCCTGGTCCCTCTTGAGGACAGTGGGGATAGGGTTGGCAC  GGCCCTCACCCCGGGGTCCTAGCCCATTCCAGGCTCCCAGCCCCCCTCAG  CAGCAGCTTGA |
| **CAT56** | CAGAGGTCCTCCAGGATCAAGGGGACCAATGATCCCACCACTGCTGAGT  CTCCCACCTCCTCCCCGGGGCAGAGGCCCAATTCGGGGAGGCCTAGGCC  CCAGGTCTGGCCCATATGGTCGTGGTTGGTGGGGGGTCAATGCCGAGCC  TCCTTTCCCTGGACCAGGCCACGGGGGTCCTTCCAGGGGAGGCTTTCAC  AAAGAACAGAGAAATCCTCGAAGGCTCAAAAGCTGGTCTCTTGTCAAG  AATACCA |
| **C2** | CCTGGAAACAAAGTAGCAGATTAGGCTGGACCAGGGCTCCTGAAGGGG  CCAGAGGCTGGGAACAAGGGGTGGGAGCTCTGCCCAGGGGATCGGGAA  TCTGGTGCGGGCCCTTCCTTACCCACACTGACCCTCCACAGTGTTCTGTC  CTCGGCATTGCTTAAGCAGTGAGCGGCTGTCAGGACCCACTGGTCGGA  GATGAGGGCCCCCCGGCAGGTCTCCTGGCTCTTGGGCTGCAGGGGGAC  AGGGAATCTCGGGGACTGCTATGTCTCTGGCTTGTGGCCACACCTCTG |
| **G6d** | CATCATTGCAATCAAGTGGAGACAGAAGTGGTGGGAGACGTGACTTATA  CGACCCACAGGGACTGCTGCGTTGGTGACCTGTGCAACGGTGCTGTGGC  GAGCACTGCAGCCCCCATGAGCATCGTGGCTGCAGCAGTCACCACGCTG  GCCTGTCTCTTGCCAGGACTGT |
| **G7c** | CTCTGCGTTTTGAGCCATACGAGGCAGTGGCTCTGGCCTCAGGAGGAGAA  GTGATTTTCACCGAAGACCAGTATATTCAGGATGTGGCGGCCATTGTTGG  GGAGAGCATGGA |
| NG36.1 | CATAGCTCTTTGGGGGACACCCTCGTAGTGAGGAGACCCTGCCCAAGGC  CAACCCTGACTCCCTGGAGACTGCTGGCCCCTCATCCCCAGCCTCTGTCA  CGGTCACTGTCGGCGATGAGGGGGCTGACACCCCTGTAGGGCCACACCA  CTCATTGGGGATGAACCTGAGAACCTTGAGGGAGATGG |
| **NG36.2** | CCATCTCCCTCAAGGTTCTCAGGTTCATCCCCAATGAGTGGTGTGGCCCC  TACAGGGGTGTCAGCCCCCTCATCGCCGACAGTGACCGTGACAGAGGCT  GGGGATGAGGGGCCAGCAGTCTCCAGGGAGTCAGGGTTGGCCTTGGGCA  GGGTCTCCTCACTACGAGGGGTGTCCCCAAAGAGCTATGAAA |
| **NOTCH4** | TAtGAGGGACAGAACtGCTCAAAGGAACCaGACGCGTGTcAATCCCAGC  cCTGTCACAACCAGGGGACCTGCACCTCCAAACCCGGAGGCTTCCACTGT  GCCTGCCCGCCGGCTTTTGTGGGGCTGCGCTGTGAGGGGGACGTGGATGA  GTGTCTGGACcGGCcCTGTCAcCCCACAGGCACTGCAGCCTGCCATTCTC  TGGCCAACGCCTTCTACTGCCAGTGTCTgCCTGGACACACAG |
| **TNFa5** | ACTTTATTTCTCGCCACTGACCAGTAGGCGGTTACAGGCATGACTCCCCTG  GGGAGCGGAGGTTCAGTGATGTAGCGACAAATCAGTCACCAAATCAGCAT  CATTTAGACAACTTG |

**Overgo primers used to amplify sheep genes in BAC clones**

Superscript R: These overgo primers were obtained from Gustafson et al. [17].

| **Gene** | **Forward Primer (5’-3’)** | **Reverse Primers (5’-3’)** |
| --- | --- | --- |
| BAT1^R^ | TGGCAGAGAACGATGTGGACAATG | TCATAGTCCAAGAGCTCATTGTCC |
| BAT1^R^ | GAAGCAGGTCATGATGTTCAGTGC | CTCTTTGCTCAAGGTAGCACTGAA |
| BAT3^R^ | CTGTTCATGACCGGAATGCCAACA | CCAACCATGACATAGCTGTTGGCA |
| BAT3 | AAGGTTCCAACCACGACATAGCTG | ATGACCGGAATGCCAACAGCTATG |
| BAT4 | CTTCTCTTCCTGCCTTTTCTCCTC | CACACTGAACCGCAGGGAGGAGAA |
| CAT56 | GGGAGGCTTTCACAAAGAACAGAG | GAGCCTTCGAGGATTTCTCTGTTC |
| C2 | AAGCAGTGAGCGGCTGTCAGGACC | TCATCTCCGACCAGTGGGTCCTGA |
| C2^R^ | AATCCATGACTCCTGCATGGCATG | CACCCCAGATTGTATGCATGCCAT |
| C4 | AGATGGTTCCTATGGGGCTTGGTT | GCTACTATCCCGATGTAACCAAGC |
| G6D | GCAATCAAGTGGAGACAGAAGTGG | TAAGTCACGTCTCCCACCACTTCT |
| G7C | TCACCGAAGACCAGTATATTCAGG | ACAATGGCCGCCACATCCTGAATA |
| HSPA1B^R^ | TGTCCATCCTGACGATCGACGACG | TTCACCTCGAAGATGCCGTCGTCG |
| MHC414 | AGCAGGAGGGGCAGGAGTATTGGG | AATCGCGTCTCCTGATCCCAATAC |
| MHC517 | GACGAGGTTTCATTTTCAGTTTGG | CACGCGGTGATTAAACCCAAACTG |
| MSH5^R^ | ATTCATGGTTCTGGCCCCACCTCT | TCTCAAGCTTCTCCAGAGAGGTGG |
| NG36 | ATCTCCCTCAAGGTTCTCAGGTTC | ACCACTCATTGGGGATGAACCTGA |
| NOTCH4 | CAGAACTGCTCAAAGGAACCAGAC | GCTGGGATTGACACGCGTCTGGTT |
| RING3 | TAGAGCCTTCATCACCACCTTGTG | CCAGTTGCAATATCTGCACAAGGT |
| TAPBP | TGCAGAGAGGCTTACAGAGCCATC | CCTGAGACATCACTCAGATGGCTC |
| TNF | ATTTCTCGCCACTGACCAGTAGGC | AGTCATGCCTGTAACCGCCTACTG |
| TNF^R^ | TACCTCATCTACTCCCAGGTCCTC | AGCCTTGGCCTTTGAAGAGGACCT |
| TNXB | CACCTACTTTCCAGGTATTCCAGC | TTTCCATCCATGCGGCGCTGGAAT |
